# 5-(4-Fluoro­phen­yl)-2-(4-methyl­phen­yl)-3-methyl­sulfanyl-1-benzo­furan

**DOI:** 10.1107/S1600536814002402

**Published:** 2014-02-08

**Authors:** Hong Dae Choi, Pil Ja Seo, Uk Lee

**Affiliations:** a Department of Chemistry, Dongeui University, San 24 Kaya-dong, Busanjin-gu, Busan 614-714, Republic of Korea; b Department of Chemistry, Pukyong National University, 599-1 Daeyeon 3-dong, Nam-gu, Busan 608-737, Republic of Korea

## Abstract

The asymmetric unit of the title compound, C_22_H_17_FOS, contains two independent mol­ecules (*A* and *B*). The dihedral angles between the benzo­furan ring systems [r.m.s. deviations of 0.026 (1), 0.004 (1) and 0.003 (1) Å, respectively, for mol­ecule *A*, and 0.002 (1), 0.004 (1) and 0.005 (1) Å for *B*] and the pendant 4-fluoro­phenyl and 4-methyl­phenyl rings are 39.48 (4) and 30.86 (5)°, respectively, for mol­ecule *A*, and 33.34 (6) and 20.99 (8)° for *B*. In the crystal, mol­ecules are linked by weak C—H⋯F and C—H⋯π inter­actions, resulting in a three-dimensional network.

## Related literature   

For background information and the crystal structures of related compounds, see: Choi *et al.* (2011*a* ▶,*b* ▶).
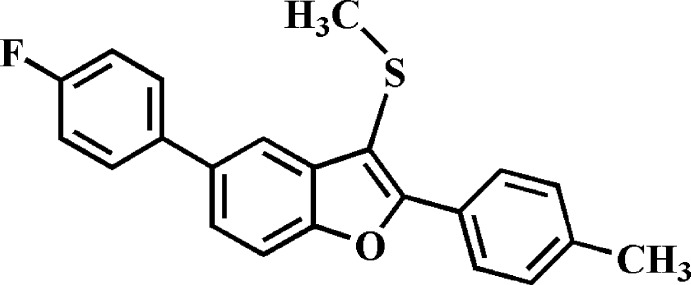


## Experimental   

### 

#### Crystal data  

C_22_H_17_FOS
*M* *_r_* = 348.42
Monoclinic, 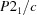

*a* = 17.897 (6) Å
*b* = 10.753 (3) Å
*c* = 17.775 (5) Å
β = 98.541 (18)°
*V* = 3382.8 (18) Å^3^
*Z* = 8
Mo *K*α radiation
μ = 0.21 mm^−1^
*T* = 173 K
0.43 × 0.36 × 0.12 mm

#### Data collection  

Bruker SMART APEXII CCD diffractometer
Absorption correction: multi-scan (*SADABS*; Bruker, 2009 ▶) *T*_min_ = 0.681, *T*_max_ = 0.746
55108 measured reflections
7388 independent reflections
5723 reflections with *I* > 2σ(*I*)
*R*_int_ = 0.051

#### Refinement  

*R*[*F* ^2^ > 2σ(*F* ^2^)] = 0.041
*wR*(*F* ^2^) = 0.114
*S* = 1.03
7388 reflections
454 parameters
H-atom parameters constrained
Δρ_max_ = 0.33 e Å^−3^
Δρ_min_ = −0.37 e Å^−3^

### 

Data collection: *APEX2* (Bruker, 2009 ▶); cell refinement: *SAINT* (Bruker, 2009 ▶); data reduction: *SAINT*; program(s) used to solve structure: *SHELXS97* (Sheldrick, 2008 ▶); program(s) used to refine structure: *SHELXL97* (Sheldrick, 2008 ▶); molecular graphics: *ORTEP-3 for Windows* (Farrugia, 2012 ▶) and *DIAMOND* (Brandenburg, 1998 ▶); software used to prepare material for publication: *SHELXL97*.

## Supplementary Material

Crystal structure: contains datablock(s) I. DOI: 10.1107/S1600536814002402/bx2454sup1.cif

Structure factors: contains datablock(s) I. DOI: 10.1107/S1600536814002402/bx2454Isup2.hkl

Supporting information file. DOI: 10.1107/S1600536814002402/bx2454Isup3.cml

CCDC reference:

Additional supporting information:  crystallographic information; 3D view; checkCIF report

## Figures and Tables

**Table 1:** Hydrogen-bond geometry (Å, °) *Cg*1 is the centroid of the C15–C20 4-methyl­phenyl ring

*D*—H⋯*A*	*D*—H	H⋯*A*	*D*⋯*A*	*D*—H⋯*A*
C28—H28⋯F2^i^	0.95	2.43	3.267 (2)	147
C44—H44*C*⋯F1^ii^	0.98	2.52	3.359 (2)	143
C32—H32⋯*Cg*1^iii^	0.95	2.69	3.465 (2)	139
C36—H36⋯*Cg*1^iv^	0.95	2.67	3.468 (2)	143

Symmetry codes: (i) 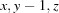; (ii) 
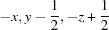
; (iii) 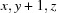; (iv) 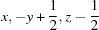.

**Fig. 1.**
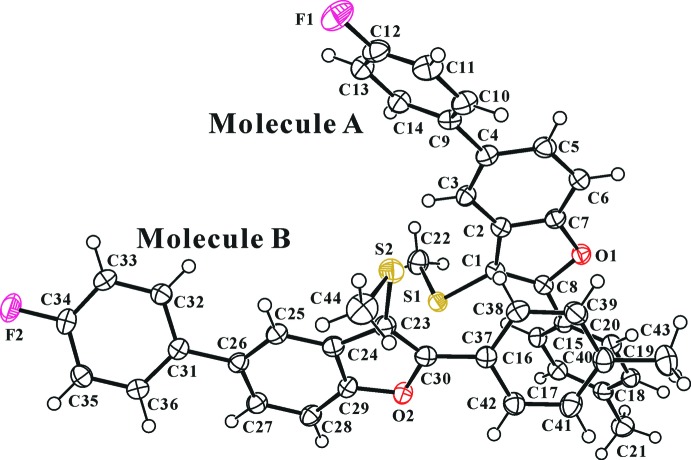
The molecular structure of the title compound with the atom numbering scheme. Displacement ellipsoids are drawn at the 50% probability level. H atoms are presented as small spheres of arbitrary radius.

**Fig. 2.**
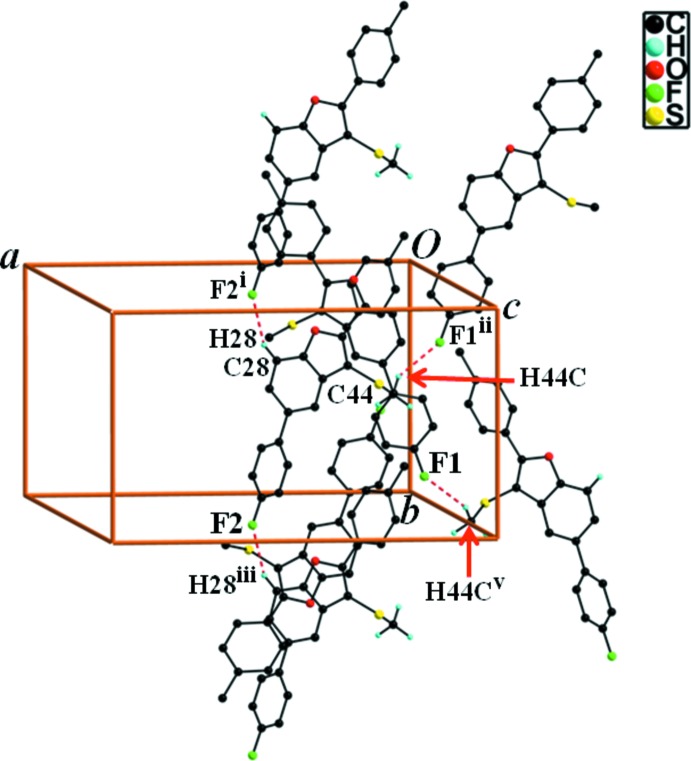
A view of the C—H···F interactions (dotted lines) in the crystal structure of the title compound. H atoms non-participating in hydrogen-bonding were omitted for clarity. [Symmetry codes: (i) x, y - 1, z (ii) - x, y - 1/2, - z + 1/2 (iii) x, y + 1, z; (v) - x, y + 1/2, - z + 1/2.]

**Fig. 3.**
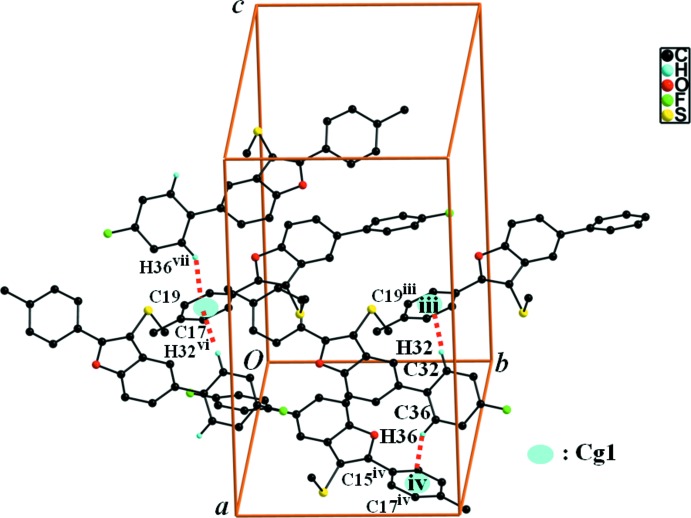
A view of the C—H···π interactions (dotted lines) in the crystal structure of the title compound. H atoms non-participating in hydrogen-bonding were omitted for clarity. [Symmetry codes: (iii) x, y + 1, z; (iv) x, - y + 1/2, z - 1/2; (vi) x, y - 1, z; (vii) x, - y + 1/2, z + 1/2.]

## supplementary crystallographic information

### 1. Comment

As a part of our continuing study of 5-(4-fluorophenyl)-3-methylsulfanyl-1-benzofuran derivatives containing 4-fluorophenyl (Choi *et al.*, 2011*a*), phenyl (Choi *et al.*, 2011*b*) substituents in 2-position, we report here the crystal structure of the title compound which crystallizes with two symmetrically independent molecules, A & B, in the asymmetric unit.

In the title molecule (Fig. 1), the benzofuran unit is essentially planar, with a mean deviation of 0.026 (1) and 0.002 (1) Å, for A and B, respectively, from the least-squares plane defined by the nine constituent atoms. The 4-fluorophenyl and 4-methylphenyl rings are essentially planar, with mean deviations of 0.004 (1) and 0.003 (1) for molecule A, and 0.004 (1) and 0.005 (1) Å for molecule B, respectively, from the least-squares plane defined by the six constituent atoms. The dihedral angles formed by the benzofuran ring system and the pendant 4-fluorophenyl and 4-methylphenyl rings are 39.48 (4) and 30.86 (5)° in molecule A, and 33.34 (6) and 20.99 (8)° in molecule B, respectively. In the crystal structure (Fig. 2 & 3), molecules are connected by weak C—H···F and C—H···π interactions (Table 2, Cg1 is the centroid of the C15–C20 4-methylphenyl ring), resulting in a three-dimensional network.

### 2. Experimental

Zinc chloride (286 mg, 2.1 mmol) was added to a stirred solution of 4-fluoro-4'-hydroxybiphenyl (376 mg, 2.0 mmol) and 2-chloro-4'-methyl-2-methylsulfanylacetophenone (429 mg, 2.0 mmol) in dichloromethane (40 mL) at room temperature, and stirring was continued at the same temperature for 1h. The reaction was quenched by the addition of water and the organic layer was separated, dried over magnesium sulfate, filtered and concentrated at reduced pressure. The residue was purified by column chromatography (hexane-benzene, 5:2 v/v) to afford the title compound as a colorless solid [yield 56%, m.p. 425–426 K; *R*_f_ = 0.56 (hexane–benzene, 5:2 v/v)]. Single crystals suitable for X-ray diffraction were prepared by slow evaporation of a solution of the title compound in carbon tetrachloride at room temperature.

### 3. Refinement

All H atoms were positioned geometrically and refined using a riding model, with C—H = 0.95 Å for aryl and 0.98 Å for methyl H atoms, respectively. *U*_iso_ (H) = 1.2*U*_eq_ (C) for aryl and 1.5*U*_eq_ (C) for methyl H atoms. The positions of methyl hydrogens were optimized rotationally.

### Crystal data

**Table:**

C_22_H_17_FOS	*F*(000) = 1456
*M**_r_* = 348.42	*D*_x_ = 1.368 Mg m^−^^3^
Monoclinic, *P*2_1_/*c*	Melting point = 425–426 K
Hall symbol: -P 2ybc	Mo *K*α radiation, λ = 0.71073 Å
*a* = 17.897 (6) Å	Cell parameters from 9590 reflections
*b* = 10.753 (3) Å	θ = 2.2–26.3°
*c* = 17.775 (5) Å	µ = 0.21 mm^−^^1^
β = 98.541 (18)°	*T* = 173 K
*V* = 3382.8 (18) Å^3^	Block, colourless
*Z* = 8	0.43 × 0.36 × 0.12 mm

### Data collection

**Table:**

Bruker SMART APEXII CCD diffractometer	7388 independent reflections
Radiation source: rotating anode	5723 reflections with *I* > 2σ(*I*)
Graphite multilayer monochromator	*R*_int_ = 0.051
Detector resolution: 10.0 pixels mm^-1^	θ_max_ = 27.0°, θ_min_ = 2.2°
φ and ω scans	*h* = −22→22
Absorption correction: multi-scan (*SADABS*; Bruker, 2009)	*k* = −13→13
*T*_min_ = 0.681, *T*_max_ = 0.746	*l* = −22→22
55108 measured reflections	

### Refinement

**Table:**

Refinement on *F*^2^	Primary atom site location: structure-invariant direct methods
Least-squares matrix: full	Secondary atom site location: difference Fourier map
*R*[*F*^2^ > 2σ(*F*^2^)] = 0.041	Hydrogen site location: difference Fourier map
*wR*(*F*^2^) = 0.114	H-atom parameters constrained
*S* = 1.03	*w* = 1/[σ^2^(*F*_o_^2^) + (0.0548*P*)^2^ + 1.2362*P*] where *P* = (*F*_o_^2^ + 2*F*_c_^2^)/3
7388 reflections	(Δ/σ)_max_ = 0.001
454 parameters	Δρ_max_ = 0.33 e Å^−^^3^
0 restraints	Δρ_min_ = −0.37 e Å^−^^3^

### Special details

**Table:**

Geometry. All esds (except the esd in the dihedral angle between two l.s. planes) are estimated using the full covariance matrix. The cell esds are taken into account individually in the estimation of esds in distances, angles and torsion angles; correlations between esds in cell parameters are only used when they are defined by crystal symmetry. An approximate (isotropic) treatment of cell esds is used for estimating esds involving l.s. planes.
Refinement. Refinement of F^2^ against ALL reflections. The weighted R-factor wR and goodness of fit S are based on F^2^, conventional R-factors R are based on F, with F set to zero for negative F^2^. The threshold expression of F^2^ > 2sigma(F^2^) is used only for calculating R-factors(gt) etc. and is not relevant to the choice of reflections for refinement. R-factors based on F^2^ are statistically about twice as large as those based on F, and R- factors based on ALL data will be even larger.

### Fractional atomic coordinates and isotropic or equivalent isotropic displacement parameters (Å^2^)

**Table:**

	*x*	*y*	*z*	*U*_iso_*/*U*_eq_	
S1	0.37299 (2)	0.21067 (4)	0.29473 (2)	0.02650 (11)	
F1	0.06413 (7)	0.84290 (10)	0.44228 (7)	0.0486 (3)	
O1	0.23646 (6)	−0.00311 (10)	0.39568 (7)	0.0263 (3)	
C1	0.30874 (9)	0.14077 (15)	0.34763 (9)	0.0237 (3)	
C2	0.25213 (9)	0.20428 (15)	0.38362 (9)	0.0241 (3)	
C3	0.23108 (9)	0.32782 (15)	0.39093 (9)	0.0252 (4)	
H3	0.2594	0.3931	0.3728	0.030*	
C4	0.16752 (9)	0.35447 (15)	0.42539 (9)	0.0262 (4)	
C5	0.12703 (10)	0.25676 (16)	0.45380 (10)	0.0290 (4)	
H5	0.0847	0.2763	0.4781	0.035*	
C6	0.14701 (10)	0.13436 (16)	0.44740 (10)	0.0288 (4)	
H6	0.1196	0.0688	0.4664	0.035*	
C7	0.20949 (9)	0.11165 (15)	0.41162 (9)	0.0253 (4)	
C8	0.29684 (9)	0.01726 (15)	0.35648 (9)	0.0241 (3)	
C9	0.14098 (9)	0.48411 (15)	0.43128 (9)	0.0261 (4)	
C10	0.06343 (10)	0.51114 (17)	0.42028 (10)	0.0316 (4)	
H10	0.0281	0.4451	0.4101	0.038*	
C11	0.03721 (10)	0.63092 (18)	0.42381 (10)	0.0352 (4)	
H11	−0.0154	0.6485	0.4158	0.042*	
C12	0.08954 (11)	0.72404 (16)	0.43927 (10)	0.0336 (4)	
C13	0.16654 (11)	0.70297 (17)	0.45160 (10)	0.0327 (4)	
H13	0.2012	0.7697	0.4629	0.039*	
C14	0.19175 (10)	0.58206 (16)	0.44707 (10)	0.0293 (4)	
H14	0.2445	0.5656	0.4548	0.035*	
C15	0.33306 (9)	−0.09598 (15)	0.33398 (9)	0.0246 (3)	
C16	0.41086 (9)	−0.10156 (16)	0.32995 (9)	0.0274 (4)	
H16	0.4412	−0.0293	0.3405	0.033*	
C17	0.44362 (10)	−0.21136 (16)	0.31081 (9)	0.0286 (4)	
H17	0.4963	−0.2135	0.3084	0.034*	
C18	0.40082 (10)	−0.31842 (15)	0.29508 (9)	0.0277 (4)	
C19	0.32341 (10)	−0.31272 (15)	0.30001 (9)	0.0277 (4)	
H19	0.2934	−0.3854	0.2901	0.033*	
C20	0.28997 (10)	−0.20375 (15)	0.31890 (9)	0.0265 (4)	
H20	0.2374	−0.2021	0.3217	0.032*	
C21	0.43623 (11)	−0.43679 (17)	0.27212 (11)	0.0371 (4)	
H21A	0.4764	−0.4628	0.3127	0.045*	
H21B	0.4577	−0.4226	0.2252	0.045*	
H21C	0.3977	−0.5020	0.2635	0.045*	
C22	0.45009 (10)	0.24833 (18)	0.36934 (11)	0.0375 (4)	
H22A	0.4314	0.3014	0.4073	0.056*	
H22B	0.4896	0.2925	0.3474	0.056*	
H22C	0.4710	0.1715	0.3936	0.056*	
S2	0.12675 (3)	0.49163 (4)	0.20081 (3)	0.03560 (13)	
F2	0.41994 (6)	1.12966 (9)	0.04363 (7)	0.0422 (3)	
O2	0.27237 (6)	0.27471 (10)	0.11261 (6)	0.0262 (3)	
C23	0.19837 (9)	0.41799 (16)	0.15898 (9)	0.0270 (4)	
C24	0.25573 (9)	0.48150 (15)	0.12453 (9)	0.0256 (4)	
C25	0.27344 (9)	0.60571 (15)	0.11360 (9)	0.0253 (3)	
H25	0.2442	0.6701	0.1315	0.030*	
C26	0.33444 (9)	0.63484 (15)	0.07614 (9)	0.0239 (3)	
C27	0.37678 (9)	0.53799 (16)	0.04921 (10)	0.0267 (4)	
H27	0.4180	0.5587	0.0235	0.032*	
C28	0.36017 (9)	0.41427 (15)	0.05910 (10)	0.0274 (4)	
H28	0.3889	0.3495	0.0410	0.033*	
C29	0.29921 (9)	0.38949 (15)	0.09688 (9)	0.0249 (4)	
C30	0.21047 (9)	0.29419 (15)	0.15035 (9)	0.0252 (3)	
C31	0.35593 (9)	0.76637 (15)	0.06618 (9)	0.0232 (3)	
C32	0.34603 (9)	0.85462 (15)	0.12153 (9)	0.0259 (4)	
H32	0.3243	0.8300	0.1649	0.031*	
C33	0.36720 (9)	0.97693 (15)	0.11432 (10)	0.0277 (4)	
H33	0.3607	1.0366	0.1522	0.033*	
C34	0.39803 (10)	1.01017 (15)	0.05080 (10)	0.0291 (4)	
C35	0.40837 (9)	0.92699 (16)	−0.00564 (10)	0.0282 (4)	
H35	0.4294	0.9529	−0.0491	0.034*	
C36	0.38730 (9)	0.80506 (15)	0.00268 (9)	0.0254 (3)	
H36	0.3942	0.7462	−0.0355	0.030*	
C37	0.17303 (9)	0.18137 (15)	0.17111 (9)	0.0251 (4)	
C38	0.12785 (10)	0.17959 (17)	0.22887 (10)	0.0312 (4)	
H38	0.1201	0.2542	0.2553	0.037*	
C39	0.09441 (10)	0.07072 (17)	0.24784 (10)	0.0335 (4)	
H39	0.0635	0.0718	0.2869	0.040*	
C40	0.10489 (10)	−0.04023 (17)	0.21115 (10)	0.0308 (4)	
C41	0.14927 (10)	−0.03773 (17)	0.15302 (11)	0.0336 (4)	
H41	0.1565	−0.1124	0.1265	0.040*	
C42	0.18297 (10)	0.07025 (16)	0.13297 (10)	0.0291 (4)	
H42	0.2130	0.0691	0.0932	0.035*	
C43	0.06869 (12)	−0.15786 (18)	0.23410 (12)	0.0441 (5)	
H43A	0.0526	−0.1461	0.2840	0.066*	
H43B	0.1052	−0.2262	0.2370	0.066*	
H43C	0.0247	−0.1779	0.1963	0.066*	
C44	0.07083 (11)	0.5533 (2)	0.11587 (12)	0.0441 (5)	
H44A	0.1030	0.6040	0.0879	0.066*	
H44B	0.0300	0.6048	0.1301	0.066*	
H44C	0.0492	0.4845	0.0836	0.066*	

### Atomic displacement parameters (Å^2^)

**Table:**

	*U*^11^	*U*^22^	*U*^33^	*U*^12^	*U*^13^	*U*^23^
S1	0.0272 (2)	0.0265 (2)	0.0265 (2)	−0.00032 (16)	0.00621 (17)	0.00148 (16)
F1	0.0554 (7)	0.0297 (6)	0.0587 (7)	0.0154 (5)	0.0019 (6)	−0.0058 (5)
O1	0.0268 (6)	0.0222 (6)	0.0312 (6)	0.0011 (5)	0.0085 (5)	0.0004 (5)
C1	0.0223 (8)	0.0247 (8)	0.0241 (8)	0.0011 (6)	0.0030 (7)	0.0004 (6)
C2	0.0218 (8)	0.0268 (8)	0.0231 (8)	0.0006 (6)	0.0015 (7)	0.0004 (6)
C3	0.0233 (8)	0.0236 (8)	0.0284 (8)	−0.0006 (6)	0.0030 (7)	−0.0016 (7)
C4	0.0235 (8)	0.0273 (9)	0.0270 (8)	0.0012 (7)	0.0007 (7)	−0.0032 (7)
C5	0.0250 (9)	0.0325 (9)	0.0304 (9)	0.0001 (7)	0.0072 (7)	−0.0045 (7)
C6	0.0288 (9)	0.0295 (9)	0.0294 (9)	−0.0024 (7)	0.0083 (7)	−0.0007 (7)
C7	0.0260 (9)	0.0239 (8)	0.0255 (8)	0.0020 (6)	0.0024 (7)	−0.0014 (6)
C8	0.0223 (8)	0.0262 (8)	0.0237 (8)	0.0010 (6)	0.0031 (7)	0.0008 (6)
C9	0.0259 (9)	0.0284 (9)	0.0241 (8)	0.0029 (7)	0.0039 (7)	−0.0034 (7)
C10	0.0257 (9)	0.0336 (10)	0.0347 (9)	0.0004 (7)	0.0012 (8)	−0.0065 (8)
C11	0.0263 (9)	0.0403 (11)	0.0377 (10)	0.0094 (8)	0.0010 (8)	−0.0056 (8)
C12	0.0430 (11)	0.0281 (9)	0.0290 (9)	0.0099 (8)	0.0032 (8)	−0.0026 (7)
C13	0.0367 (10)	0.0283 (9)	0.0332 (9)	−0.0023 (8)	0.0051 (8)	−0.0024 (7)
C14	0.0259 (9)	0.0297 (9)	0.0322 (9)	0.0016 (7)	0.0044 (7)	−0.0006 (7)
C15	0.0276 (9)	0.0237 (8)	0.0226 (8)	0.0024 (7)	0.0038 (7)	0.0025 (6)
C16	0.0284 (9)	0.0252 (8)	0.0288 (9)	−0.0007 (7)	0.0045 (7)	0.0014 (7)
C17	0.0271 (9)	0.0305 (9)	0.0286 (9)	0.0037 (7)	0.0056 (7)	0.0025 (7)
C18	0.0352 (9)	0.0248 (8)	0.0233 (8)	0.0058 (7)	0.0050 (7)	0.0031 (7)
C19	0.0350 (10)	0.0237 (8)	0.0240 (8)	−0.0019 (7)	0.0027 (7)	0.0010 (7)
C20	0.0256 (8)	0.0274 (9)	0.0263 (8)	−0.0003 (7)	0.0033 (7)	0.0011 (7)
C21	0.0443 (11)	0.0298 (10)	0.0382 (10)	0.0089 (8)	0.0093 (9)	−0.0009 (8)
C22	0.0319 (10)	0.0365 (10)	0.0427 (11)	−0.0060 (8)	0.0009 (8)	0.0003 (8)
S2	0.0374 (3)	0.0324 (3)	0.0401 (3)	0.00336 (19)	0.0163 (2)	−0.00116 (19)
F2	0.0504 (7)	0.0216 (5)	0.0580 (7)	−0.0052 (5)	0.0193 (6)	−0.0003 (5)
O2	0.0250 (6)	0.0221 (6)	0.0318 (6)	−0.0013 (4)	0.0056 (5)	−0.0007 (5)
C23	0.0267 (9)	0.0274 (9)	0.0271 (8)	−0.0001 (7)	0.0048 (7)	−0.0018 (7)
C24	0.0245 (8)	0.0268 (9)	0.0251 (8)	0.0006 (7)	0.0023 (7)	−0.0012 (7)
C25	0.0252 (8)	0.0237 (8)	0.0269 (8)	0.0012 (6)	0.0036 (7)	−0.0022 (7)
C26	0.0240 (8)	0.0233 (8)	0.0230 (8)	−0.0007 (6)	−0.0007 (7)	−0.0014 (6)
C27	0.0239 (8)	0.0271 (9)	0.0295 (9)	−0.0015 (7)	0.0050 (7)	−0.0019 (7)
C28	0.0257 (9)	0.0247 (9)	0.0321 (9)	0.0026 (7)	0.0054 (7)	−0.0035 (7)
C29	0.0250 (8)	0.0218 (8)	0.0270 (8)	−0.0013 (6)	0.0010 (7)	−0.0010 (6)
C30	0.0215 (8)	0.0294 (9)	0.0243 (8)	−0.0003 (7)	0.0019 (7)	−0.0022 (7)
C31	0.0191 (8)	0.0232 (8)	0.0259 (8)	0.0007 (6)	−0.0010 (6)	−0.0001 (6)
C32	0.0257 (9)	0.0262 (9)	0.0259 (8)	0.0012 (7)	0.0040 (7)	0.0002 (7)
C33	0.0282 (9)	0.0243 (8)	0.0303 (9)	0.0019 (7)	0.0038 (7)	−0.0054 (7)
C34	0.0269 (9)	0.0213 (8)	0.0387 (10)	−0.0021 (7)	0.0033 (8)	0.0021 (7)
C35	0.0261 (9)	0.0307 (9)	0.0283 (9)	−0.0012 (7)	0.0059 (7)	0.0024 (7)
C36	0.0240 (8)	0.0269 (8)	0.0250 (8)	0.0012 (7)	0.0026 (7)	−0.0027 (7)
C37	0.0226 (8)	0.0267 (8)	0.0248 (8)	−0.0005 (6)	−0.0005 (7)	0.0014 (7)
C38	0.0336 (10)	0.0313 (9)	0.0282 (9)	−0.0034 (7)	0.0028 (8)	−0.0047 (7)
C39	0.0337 (10)	0.0392 (10)	0.0286 (9)	−0.0036 (8)	0.0075 (8)	0.0011 (8)
C40	0.0300 (9)	0.0298 (9)	0.0316 (9)	−0.0020 (7)	0.0005 (7)	0.0068 (7)
C41	0.0346 (10)	0.0250 (9)	0.0413 (10)	0.0013 (7)	0.0063 (8)	−0.0005 (8)
C42	0.0282 (9)	0.0280 (9)	0.0320 (9)	0.0012 (7)	0.0076 (7)	0.0010 (7)
C43	0.0476 (12)	0.0358 (11)	0.0505 (12)	−0.0039 (9)	0.0120 (10)	0.0113 (9)
C44	0.0317 (10)	0.0470 (12)	0.0538 (13)	0.0058 (9)	0.0065 (9)	−0.0034 (10)

### Geometric parameters (Å, º)

**Table:**

S1—C1	1.7586 (17)	S2—C23	1.7623 (18)
S1—C22	1.8119 (19)	S2—C44	1.809 (2)
F1—C12	1.360 (2)	F2—C34	1.3550 (19)
O1—C7	1.3699 (19)	O2—C29	1.3683 (19)
O1—C8	1.388 (2)	O2—C30	1.393 (2)
C1—C8	1.358 (2)	C23—C30	1.361 (2)
C1—C2	1.447 (2)	C23—C24	1.443 (2)
C2—C7	1.391 (2)	C24—C29	1.393 (2)
C2—C3	1.392 (2)	C24—C25	1.393 (2)
C3—C4	1.399 (2)	C25—C26	1.396 (2)
C3—H3	0.9500	C25—H25	0.9500
C4—C5	1.412 (2)	C26—C27	1.413 (2)
C4—C9	1.481 (2)	C26—C31	1.483 (2)
C5—C6	1.373 (2)	C27—C28	1.380 (2)
C5—H5	0.9500	C27—H27	0.9500
C6—C7	1.388 (2)	C28—C29	1.389 (2)
C6—H6	0.9500	C28—H28	0.9500
C8—C15	1.463 (2)	C30—C37	1.460 (2)
C9—C14	1.392 (2)	C31—C36	1.397 (2)
C9—C10	1.403 (2)	C31—C32	1.397 (2)
C10—C11	1.375 (3)	C32—C33	1.380 (2)
C10—H10	0.9500	C32—H32	0.9500
C11—C12	1.371 (3)	C33—C34	1.375 (3)
C11—H11	0.9500	C33—H33	0.9500
C12—C13	1.382 (3)	C34—C35	1.377 (2)
C13—C14	1.382 (2)	C35—C36	1.378 (2)
C13—H13	0.9500	C35—H35	0.9500
C14—H14	0.9500	C36—H36	0.9500
C15—C20	1.396 (2)	C37—C42	1.398 (2)
C15—C16	1.406 (2)	C37—C38	1.398 (2)
C16—C17	1.383 (2)	C38—C39	1.379 (3)
C16—H16	0.9500	C38—H38	0.9500
C17—C18	1.388 (2)	C39—C40	1.386 (3)
C17—H17	0.9500	C39—H39	0.9500
C18—C19	1.402 (3)	C40—C41	1.394 (3)
C18—C21	1.505 (2)	C40—C43	1.505 (3)
C19—C20	1.380 (2)	C41—C42	1.379 (2)
C19—H19	0.9500	C41—H41	0.9500
C20—H20	0.9500	C42—H42	0.9500
C21—H21A	0.9800	C43—H43A	0.9800
C21—H21B	0.9800	C43—H43B	0.9800
C21—H21C	0.9800	C43—H43C	0.9800
C22—H22A	0.9800	C44—H44A	0.9800
C22—H22B	0.9800	C44—H44B	0.9800
C22—H22C	0.9800	C44—H44C	0.9800
			
C1—S1—C22	101.05 (9)	C23—S2—C44	99.18 (9)
C7—O1—C8	106.64 (12)	C29—O2—C30	106.93 (12)
C8—C1—C2	106.15 (14)	C30—C23—C24	106.25 (15)
C8—C1—S1	127.32 (13)	C30—C23—S2	128.68 (14)
C2—C1—S1	126.22 (12)	C24—C23—S2	125.05 (13)
C7—C2—C3	118.64 (15)	C29—C24—C25	118.77 (16)
C7—C2—C1	106.11 (14)	C29—C24—C23	106.47 (15)
C3—C2—C1	135.11 (16)	C25—C24—C23	134.74 (16)
C2—C3—C4	119.02 (16)	C24—C25—C26	119.46 (15)
C2—C3—H3	120.5	C24—C25—H25	120.3
C4—C3—H3	120.5	C26—C25—H25	120.3
C3—C4—C5	119.87 (15)	C25—C26—C27	119.54 (15)
C3—C4—C9	120.91 (15)	C25—C26—C31	120.39 (15)
C5—C4—C9	119.21 (15)	C27—C26—C31	120.05 (15)
C6—C5—C4	122.01 (16)	C28—C27—C26	122.06 (16)
C6—C5—H5	119.0	C28—C27—H27	119.0
C4—C5—H5	119.0	C26—C27—H27	119.0
C5—C6—C7	116.37 (16)	C27—C28—C29	116.49 (15)
C5—C6—H6	121.8	C27—C28—H28	121.8
C7—C6—H6	121.8	C29—C28—H28	121.8
O1—C7—C6	125.87 (15)	O2—C29—C28	126.63 (15)
O1—C7—C2	110.01 (14)	O2—C29—C24	109.70 (14)
C6—C7—C2	124.07 (16)	C28—C29—C24	123.67 (15)
C1—C8—O1	111.09 (14)	C23—C30—O2	110.64 (15)
C1—C8—C15	134.35 (16)	C23—C30—C37	134.24 (16)
O1—C8—C15	114.57 (14)	O2—C30—C37	115.11 (14)
C14—C9—C10	118.29 (16)	C36—C31—C32	118.37 (15)
C14—C9—C4	121.31 (15)	C36—C31—C26	121.54 (15)
C10—C9—C4	120.40 (15)	C32—C31—C26	120.09 (15)
C11—C10—C9	121.66 (17)	C33—C32—C31	121.11 (16)
C11—C10—H10	119.2	C33—C32—H32	119.4
C9—C10—H10	119.2	C31—C32—H32	119.4
C12—C11—C10	117.74 (17)	C34—C33—C32	118.24 (16)
C12—C11—H11	121.1	C34—C33—H33	120.9
C10—C11—H11	121.1	C32—C33—H33	120.9
F1—C12—C11	118.15 (17)	F2—C34—C33	118.73 (15)
F1—C12—C13	118.64 (17)	F2—C34—C35	118.38 (16)
C11—C12—C13	123.21 (17)	C33—C34—C35	122.89 (16)
C12—C13—C14	118.13 (17)	C34—C35—C36	118.11 (16)
C12—C13—H13	120.9	C34—C35—H35	120.9
C14—C13—H13	120.9	C36—C35—H35	120.9
C13—C14—C9	120.95 (16)	C35—C36—C31	121.29 (16)
C13—C14—H14	119.5	C35—C36—H36	119.4
C9—C14—H14	119.5	C31—C36—H36	119.4
C20—C15—C16	118.55 (15)	C42—C37—C38	118.26 (16)
C20—C15—C8	119.27 (15)	C42—C37—C30	119.43 (15)
C16—C15—C8	122.13 (15)	C38—C37—C30	122.30 (15)
C17—C16—C15	120.54 (16)	C39—C38—C37	120.69 (17)
C17—C16—H16	119.7	C39—C38—H38	119.7
C15—C16—H16	119.7	C37—C38—H38	119.7
C16—C17—C18	121.08 (16)	C38—C39—C40	121.44 (18)
C16—C17—H17	119.5	C38—C39—H39	119.3
C18—C17—H17	119.5	C40—C39—H39	119.3
C17—C18—C19	118.15 (15)	C39—C40—C41	117.66 (17)
C17—C18—C21	120.93 (17)	C39—C40—C43	120.14 (17)
C19—C18—C21	120.91 (16)	C41—C40—C43	122.20 (17)
C20—C19—C18	121.38 (16)	C42—C41—C40	121.80 (17)
C20—C19—H19	119.3	C42—C41—H41	119.1
C18—C19—H19	119.3	C40—C41—H41	119.1
C19—C20—C15	120.30 (16)	C41—C42—C37	120.14 (17)
C19—C20—H20	119.9	C41—C42—H42	119.9
C15—C20—H20	119.9	C37—C42—H42	119.9
C18—C21—H21A	109.5	C40—C43—H43A	109.5
C18—C21—H21B	109.5	C40—C43—H43B	109.5
H21A—C21—H21B	109.5	H43A—C43—H43B	109.5
C18—C21—H21C	109.5	C40—C43—H43C	109.5
H21A—C21—H21C	109.5	H43A—C43—H43C	109.5
H21B—C21—H21C	109.5	H43B—C43—H43C	109.5
S1—C22—H22A	109.5	S2—C44—H44A	109.5
S1—C22—H22B	109.5	S2—C44—H44B	109.5
H22A—C22—H22B	109.5	H44A—C44—H44B	109.5
S1—C22—H22C	109.5	S2—C44—H44C	109.5
H22A—C22—H22C	109.5	H44A—C44—H44C	109.5
H22B—C22—H22C	109.5	H44B—C44—H44C	109.5
			
C22—S1—C1—C8	−99.82 (16)	C44—S2—C23—C30	−108.34 (17)
C22—S1—C1—C2	87.45 (15)	C44—S2—C23—C24	69.81 (16)
C8—C1—C2—C7	−0.26 (17)	C30—C23—C24—C29	0.09 (18)
S1—C1—C2—C7	173.73 (12)	S2—C23—C24—C29	−178.40 (12)
C8—C1—C2—C3	−175.84 (18)	C30—C23—C24—C25	178.91 (18)
S1—C1—C2—C3	−1.8 (3)	S2—C23—C24—C25	0.4 (3)
C7—C2—C3—C4	−0.6 (2)	C29—C24—C25—C26	−0.7 (2)
C1—C2—C3—C4	174.53 (16)	C23—C24—C25—C26	−179.37 (17)
C2—C3—C4—C5	1.6 (2)	C24—C25—C26—C27	0.6 (2)
C2—C3—C4—C9	−177.22 (15)	C24—C25—C26—C31	−177.91 (14)
C3—C4—C5—C6	−1.4 (2)	C25—C26—C27—C28	−0.4 (2)
C9—C4—C5—C6	177.43 (16)	C31—C26—C27—C28	178.15 (15)
C4—C5—C6—C7	0.2 (2)	C26—C27—C28—C29	0.2 (2)
C8—O1—C7—C6	177.01 (16)	C30—O2—C29—C28	−179.38 (16)
C8—O1—C7—C2	−0.41 (17)	C30—O2—C29—C24	0.46 (17)
C5—C6—C7—O1	−176.25 (15)	C27—C28—C29—O2	179.60 (15)
C5—C6—C7—C2	0.8 (2)	C27—C28—C29—C24	−0.2 (2)
C3—C2—C7—O1	176.86 (14)	C25—C24—C29—O2	−179.39 (14)
C1—C2—C7—O1	0.41 (17)	C23—C24—C29—O2	−0.34 (17)
C3—C2—C7—C6	−0.6 (2)	C25—C24—C29—C28	0.5 (2)
C1—C2—C7—C6	−177.06 (15)	C23—C24—C29—C28	179.51 (15)
C2—C1—C8—O1	0.02 (18)	C24—C23—C30—O2	0.20 (18)
S1—C1—C8—O1	−173.89 (11)	S2—C23—C30—O2	178.61 (12)
C2—C1—C8—C15	179.88 (17)	C24—C23—C30—C37	−179.22 (17)
S1—C1—C8—C15	6.0 (3)	S2—C23—C30—C37	−0.8 (3)
C7—O1—C8—C1	0.24 (18)	C29—O2—C30—C23	−0.41 (18)
C7—O1—C8—C15	−179.66 (13)	C29—O2—C30—C37	179.13 (13)
C3—C4—C9—C14	−38.7 (2)	C25—C26—C31—C36	−148.12 (16)
C5—C4—C9—C14	142.52 (17)	C27—C26—C31—C36	33.3 (2)
C3—C4—C9—C10	140.75 (17)	C25—C26—C31—C32	32.8 (2)
C5—C4—C9—C10	−38.1 (2)	C27—C26—C31—C32	−145.73 (16)
C14—C9—C10—C11	0.8 (3)	C36—C31—C32—C33	−0.6 (2)
C4—C9—C10—C11	−178.60 (17)	C26—C31—C32—C33	178.51 (15)
C9—C10—C11—C12	−0.6 (3)	C31—C32—C33—C34	0.4 (2)
C10—C11—C12—F1	179.49 (16)	C32—C33—C34—F2	−179.14 (15)
C10—C11—C12—C13	−0.3 (3)	C32—C33—C34—C35	0.2 (3)
F1—C12—C13—C14	−178.87 (16)	F2—C34—C35—C36	178.78 (15)
C11—C12—C13—C14	0.9 (3)	C33—C34—C35—C36	−0.5 (3)
C12—C13—C14—C9	−0.6 (3)	C34—C35—C36—C31	0.3 (2)
C10—C9—C14—C13	−0.2 (3)	C32—C31—C36—C35	0.2 (2)
C4—C9—C14—C13	179.24 (16)	C26—C31—C36—C35	−178.87 (15)
C1—C8—C15—C20	−149.90 (19)	C23—C30—C37—C42	158.75 (18)
O1—C8—C15—C20	30.0 (2)	O2—C30—C37—C42	−20.6 (2)
C1—C8—C15—C16	32.7 (3)	C23—C30—C37—C38	−22.0 (3)
O1—C8—C15—C16	−147.39 (15)	O2—C30—C37—C38	158.57 (15)
C20—C15—C16—C17	0.5 (2)	C42—C37—C38—C39	0.4 (2)
C8—C15—C16—C17	177.92 (15)	C30—C37—C38—C39	−178.86 (16)
C15—C16—C17—C18	0.1 (3)	C37—C38—C39—C40	0.6 (3)
C16—C17—C18—C19	−0.8 (2)	C38—C39—C40—C41	−1.3 (3)
C16—C17—C18—C21	178.26 (16)	C38—C39—C40—C43	178.95 (17)
C17—C18—C19—C20	0.9 (2)	C39—C40—C41—C42	1.1 (3)
C21—C18—C19—C20	−178.14 (16)	C43—C40—C41—C42	−179.22 (17)
C18—C19—C20—C15	−0.3 (3)	C40—C41—C42—C37	−0.1 (3)
C16—C15—C20—C19	−0.4 (2)	C38—C37—C42—C41	−0.6 (2)
C8—C15—C20—C19	−177.87 (15)	C30—C37—C42—C41	178.63 (15)

### Hydrogen-bond geometry (Å, º)

Cg1 is the centroid of the C15–C20 4-methylphenyl ring

**Table:**

*D*—H···*A*	*D*—H	H···*A*	*D*···*A*	*D*—H···*A*
C28—H28···F2^i^	0.95	2.43	3.267 (2)	147
C44—H44*C*···F1^ii^	0.98	2.52	3.359 (2)	143
C32—H32···*Cg*1^iii^	0.95	2.69	3.465 (2)	139
C36—H36···*Cg*1^iv^	0.95	2.67	3.468 (2)	143

Symmetry codes: (i) *x*, *y*−1, *z*; (ii) −*x*, *y*−1/2, −*z*+1/2; (iii) *x*, *y*+1, *z*; (iv) *x*, −*y*+1/2, *z*−1/2.
